# Supplementary material for: Differentially expressed genes in orbital adipose/connective tissue of thyroid-associated orbitopathy
Source: PeerJ. 2023 Dec 18;11:e16569. doi: 10.7717/peerj.16569 (PMC10734407; doi:10.7717/peerj.16569)
Supplement: Supplemental Information 1 [file peerj-11-16569-s001.docx]

**Differentially expressed genes in orbital adipose/connective tissue of thyroid-associated orbitopathy**

Yan Wang^1^, Yanqiu Liu^1^, Jiping Cai^1^, Tianyi Zong^1^ , Ziyin Zhang^1^ , Tianhua Xie^1^ , Tong Mu^1^ , Meili Wu^2^ , Qian Yang^1,2^ , Yangningzhi Wang^1^ , Xiaolu Wang^2,^ *, Yong Yao^1,^ *

^1^ Department of Ophthalmology, The Affiliated Wuxi People's Hospital of Nanjing Medical University, Wuxi People's Hospital, Wuxi Medical Center, Nanjing Medical University, 299 Qingyang Road, Wuxi, Jiangsu 214023, People’s Republic of China

^2^ Center of Clinical Research, The Affiliated Wuxi People's Hospital of Nanjing Medical University, Wuxi People's Hospital, Wuxi Medical Center, Nanjing Medical University, 299 Qingyang Road, Wuxi, Jiangsu 214023, People’s Republic of China

Corresponding Author:

Yong Yao^1^

299 Qingyang Road, Wuxi, Jiangsu, 214023, People’s Republic of China

E–mail: yongyao@njmu.edu.cn

Xiaolu Wang^2^

299 Qingyang Road, Wuxi, Jiangsu, 214023, People’s Republic of China

E-mail: xlwang@njmu.edu.cn

**Supplementary Figure 1. The correlation heatmap between PCs with patient demographics.**

**Supplementary Figure 2. Qualification and analysis of identified genes from RNA sequencing data.**

(A) Violin plot of transcript FPKM values in the TAO and control groups.

(B) Box-plots of normalized FPKM values in the TAO and control groups.

(C) Q-Q plots of normalized FPKM values.

(D) Principal component analysis.

**Supplementary Figure 3. Histology changes in the orbital muscle tissue of TAO patients and control individuals**

(A and B) Immunohistochemistry for CD45 (A) and ICAM1 (B) was performed on orbital muscle tissue; scale bar, 50 μm.

(C) Immunofluorescence detection of α-SMA or FN (green) in orbital adipose tissue from TAO patients and control individuals; scale bar, 50 μm. FN: fibronectin.

**Supplementary Tables**

**Supplementary Table 1** The Reads Quality Date of RNA sequencing.

| **Sample Name** | **Clean Reads** | **Clean Bases(bp)** | **Clean N(%)** | **Clean GC(%)** | **Clean Q20(%)** | **Clean Q30(%)** | **rRNA Ratio(%)** |
| --- | --- | --- | --- | --- | --- | --- | --- |
| CON-1 | 43472508 | 6520876200 | 0.00;0.00 | 50.01;50.09 | 96.90;95.80 | 91.96;89.74 | 0.17 |
| CON-2  CON-3  TAO-1  TAO-2  TAO-3 | 40171144  42674616  43466170  48970308  49655858 | 6025671600  6401192400  6519925500  7345546200  7448378700 | 0.00;0.00  0.00;0.00  0.00;0.00  0.00;0.00  0.00;0.00 | 51.12;51.02  50.50;50.45  50.64;50.54  49.89;49.93  50.26;50.21 | 97.36;96.46  97.27;96.22  97.44;95.90  96.95;95.77  97.36;96.67 | 93.21;91.31  92.93;90.74  93.35;90.00  91.99;89.57  93.15;91.69 | 0.17  0.14  0.14  0.12  0.17 |

**Supplementary Table 2** Primer sequences and conditions for conventional RT-PCR

| **Genes** | **Forward Primer (5’-3’)** | **Reverse Primer (5’-3’)** |
| --- | --- | --- |
| MAB21L1 | GGGATCGTCACCTTGAACTG | GCAGGCAGGAGATAAGTTGC |
| TPO | ACACAGGCAAATCCGAAATC | GAGTGGTGTTTCCAGGGTTC |
| POU3F3 | GTTCTCGCAGACCACCATCT | CCTTGACGCTCACCTCGATA |
| SIGLEC14 | CTCCTCACCTATGGCCTCA | GTGGGATGTGAGCTTCCAGA |
| CLVS2 | CGGGCTAGGAAGTTTCATCA | CCTGCCATAGTGGTCCAGAT |
| PIK3C2G | CAAGGGAATCCATGCTCACT | TGGCTGACTTACATCCCACA |
| RSPO1 | TGCTCTGACGGTGGTAAACA | TTTGGGTAACATGCCAATCA |
| ALPL | CCACGTCTTCACATTTGGTG | GACTGCGCCTGGTAGTTGTT |
| CP | GAATCAGCCGGGTCTCACTA | CTTCATGCCGCCTGTGTAAT |
| GPX3 | GGCACCATTTACGAGTACGG | CCAGAATGACCAGACCGAAT |
| BPIFB4 | GTCCTCAGGGTGACGAAAGA | CCTCGGACATGGAAGTCATT |
| KCNK10 | AGTTGTACCCACGACATCCTC | GGAAGGGTCTCATAAGCAATG |
| SERPINA3 | AGTCACCAATCCCAAGCAAG | CCCTGTGCATGTGAGAGCTA |
| ACAN | GAACGACAGGACCATCGAA | GGTGGTAATTGCAGGGAACAT |
| AGT | GGCCAGCAGCAGATAACAAC | AACAGTAGTCCCGCGCTAAA |
| OLFM4 | CCAACACGATTGCTGTGACT | ACCATGAAGGCGTTAGAAGC |

**Supplementary Table 3** The degree and Closeness Centrality.

| **Number** | **Gene Name** | **Degree** | **ClosenessCentrality** |
| --- | --- | --- | --- |
| 1 | **CXCL8** | 20 | 0.332278481 |
| 2 | TLR2 | 16 | 0.31626506 |
| 3 | **CCL4** | 12 | 0.3 |
| 4 | AGT | 11 | 0.288461538 |
| 5 | SAA1 | 10 | 0.283783784 |
| 6 | CXCR4 | 10 | 0.290858726 |
| 7 | HIF1A | 10 | 0.296610169 |
| 8 | ACAN | 10 | 0.3115727 |
| 9 | **FPR1** | 9 | 0.276315789 |
| 10 | PLEK | 9 | 0.28150134 |
| 11 | FCGR1A | 9 | 0.300859599 |
| 12 | NTS | 7 | 0.250596659 |
| 13 | **S100A9** | 7 | 0.28150134 |
| 14 | TBX1 | 6 | 0.160305344 |
| 15 | MEIS1 | 6 | 0.220125786 |
| 16 | EDN3 | 6 | 0.242494226 |
| 17 | CP | 6 | 0.265151515 |
| 18 | ZIC1 | 5 | 0.160305344 |
| 19 | SLC30A8 | 5 | 0.222929936 |
| 20 | NGFR | 5 | 0.234375 |
| 21 | **COMP** | 5 | 0.256097561 |
| 22 | ADAMTS9 | 5 | 0.260545906 |
| 23 | MCL1 | 5 | 0.265822785 |
| 24 | BCL2A1 | 5 | 0.27486911 |
| 25 | HMOX1 | 5 | 0.27486911 |
| 26 | VCAN | 5 | 0.283783784 |
| 27 | C4A | 5 | 0.29494382 |
| 28 | PTHLH | 5 | 0.296610169 |
| 29 | MPZL2 | 4 | 0.159332322 |
| 30 | POU3F3 | 4 | 0.159574468 |
| 31 | NR2F1 | 4 | 0.184859155 |
| 32 | HOXC9 | 4 | 0.185512367 |
| 33 | GFRA1 | 4 | 0.206692913 |
| 34 | PRKAR2B | 4 | 0.244755245 |
| 35 | CDH11 | 4 | 0.25 |
| 36 | GIG25 | 4 | 0.253012048 |
| 37 | PI3 | 4 | 0.275590551 |
| 38 | IHH | 4 | 0.277044855 |
| 39 | RNASE2 | 4 | 0.280748663 |
| 40 | GATA6 | 3 | 0.159332322 |
| 41 | RSPO1 | 3 | 0.183887916 |
| 42 | TBX3 | 3 | 0.183887916 |
| 43 | PCSK1 | 3 | 0.185185185 |
| 44 | COL4A6 | 3 | 0.195895522 |
| 45 | PKP2 | 3 | 0.217391304 |
| 46 | ZIC2 | 3 | 0.219206681 |
| 47 | WNT5B | 3 | 0.221987315 |
| 48 | EDNRB | 3 | 0.22826087 |
| 49 | ACE2 | 3 | 0.234899329 |
| 50 | RUNX1 | 3 | 0.239726027 |
| 51 | **CDH4** | 3 | 0.2402746 |
| 52 | COL9A3 | 3 | 0.240825688 |
| 53 | **PPL** | 3 | 0.241935484 |
| 54 | ADAMTS18 | 3 | 0.244186047 |
| 55 | CDON | 3 | 0.244186047 |
| 56 | VNN2 | 3 | 0.245901639 |
| 57 | FGF1 | 3 | 0.260545906 |
| 58 | IL1R2 | 3 | 0.261845387 |
| 59 | MSR1 | 3 | 0.2625 |
| 60 | CR1 | 3 | 0.266497462 |
| 61 | 5-Sep | 2 | 0.138522427 |
| 62 | GP1BB | 2 | 0.138522427 |
| 63 | T | 2 | 0.138888889 |
| 64 | OLFM4 | 2 | 0.156017831 |
| 65 | SCG5 | 2 | 0.156950673 |
| 66 | ELL2 | 2 | 0.156950673 |
| 67 | **ALX1** | 2 | 0.157421289 |
| 68 | MAB21L1 | 2 | 0.181975737 |
| 69 | **SHC3** | 2 | 0.191605839 |
| 70 | TNS4 | 2 | 0.195895522 |
| 71 | FOSB | 2 | 0.202702703 |
| 72 | **FAP** | 2 | 0.217842324 |
| 73 | PIK3C2G | 2 | 0.21875 |
| 74 | **BMF** | 2 | 0.221987315 |
| 75 | SLC39A14 | 2 | 0.227765727 |
| 76 | PFKFB3 | 2 | 0.230263158 |
| 77 | HIF3A | 2 | 0.230263158 |
| 78 | MYH2 | 2 | 0.233853007 |
| 79 | ALPL | 2 | 0.2402746 |
| 80 | LCP1 | 2 | 0.240825688 |
| 81 | ADCYAP1 | 2 | 0.240825688 |
| 82 | **NFKBIZ** | 2 | 0.252403846 |
| 83 | ZMYND8 | 2 | 0.254854369 |
| 84 | IL16 | 2 | 0.257985258 |
| 85 | KCNA1 | 2 | 0.75 |
| 86 | RPS26 | 2 | 0.75 |
| 87 | **CPXM1** | 2 | 1 |
| 88 | CD177 | 1 | 0.135135135 |
| 89 | PCSK6 | 1 | 0.135834411 |
| 90 | NABP1 | 1 | 0.135834411 |
| 91 | **COCH** | 1 | 0.137614679 |
| 92 | CYP19A1 | 1 | 0.155555556 |
| 93 | **COL6A6** | 1 | 0.1640625 |
| 94 | DUSP2 | 1 | 0.168810289 |
| 95 | MAPK4 | 1 | 0.178875639 |
| 96 | FOXD1 | 1 | 0.180102916 |
| 97 | SFRP4 | 1 | 0.181975737 |
| 98 | TPO | 1 | 0.182608696 |
| 99 | ADRA2A | 1 | 0.182608696 |
| 100 | PNCK | 1 | 0.1875 |
| 101 | **HMCN2** | 1 | 0.1875 |
| 102 | PDE4D | 1 | 0.196998124 |
| 103 | GPX3 | 1 | 0.216049383 |
| 104 | SLA | 1 | 0.220125786 |
| 105 | PHLDA1 | 1 | 0.220125786 |
| 106 | F13A1 | 1 | 0.220125786 |
| 107 | ARAP2 | 1 | 0.220125786 |
| 108 | S1PR3 | 1 | 0.225806452 |
| 109 | SIGLEC14 | 1 | 0.240825688 |
| 110 | KCNK10 | 1 | 0.5 |
| 111 | EIF3CL | 1 | 0.5 |
| 112 | TMEM176A | 1 | 0.666666667 |
| 113 | MMRN1 | 1 | 0.666666667 |
| 114 | XIRP1 | 1 | 1 |
| 115 | SUSD5 | 1 | 1 |
| 116 | SLC7A5 | 1 | 1 |
| 117 | SLC6A15 | 1 | 1 |
| 118 | SAMD4A | 1 | 1 |
| 119 | NWD2 | 1 | 1 |
| 120 | PAPPA2 | 1 | 1 |
| 121 | **NELL2** | 1 | 1 |
| 122 | BAIAP3 | 1 | 1 |
| 123 | BAIAP2 | 1 | 1 |
